# Empowerment through health self-testing apps? Revisiting empowerment as a process

**DOI:** 10.1007/s11019-022-10132-w

**Published:** 2023-01-02

**Authors:** Alexandra Kapeller, Iris Loosman

**Affiliations:** 1 Department of Thematic Studies – Technology and Social Change, Linköping University, Linköping, Sweden; 2 Department of Philosophy and Ethics, Eindhoven University of Technology, Eindhoven, The Netherlands

**Keywords:** Self-testing apps, mHealth, Digital health technologies, Empowerment, Bioethics

## Abstract

Empowerment, an already central concept in public health, has gained additional relevance through the expansion of mobile health (mHealth). Especially direct-to-consumer self-testing app companies mobilise the term to advertise their products, which allow users to self-test for various medical conditions independent of healthcare professionals. This article first demonstrates the absence of empowerment conceptualisations in the context of self-testing apps by engaging with empowerment literature. It then contrasts the service these apps provide with two widely cited empowerment definitions by the WHO, which describe the term as a process that, broadly, leads to knowledge and control of health decisions. We conclude that self-testing apps can only partly empower their users, as they, we argue, do not provide the type of knowledge and control the WHO definitions describe. More importantly, we observe that this shortcoming stems from the fact that in the literature on mHealth and in self-testing marketing, empowerment is understood as a goal rather than a process. This characterises a shift in the meaning of empowerment in the context of self-testing and mHealth, one that reveals a lack of awareness for relational and contextual factors that contribute to empowerment. We argue that returning to a process-understanding of empowerment helps to identify these apps’ deficits, and we conclude the article by briefly suggesting several strategies to increase self-testing apps’ empowerment function.

## Introduction

The term empowerment has gained new traction in healthcare and public health through the expansion of mobile health (mHealth), where promises of empowerment are frequently used to sell mHealth apps. Empowerment, with its roots in political movements and its application in a wide range of contexts, e.g. law, academia, politics, and business (Gibson 1991), is difficult to define (Tengland 2007, 2008; Agner and Braun 2018; Morley and Floridi 2020), and easy to create false expectations with (Nordgren 2013).

In this article, we problematise the mobilisation of this concept in the new context of *self-testing apps*. These mHealth apps are an emerging technology that allow smartphone and tablet users to test themselves for medical conditions. Such apps have become increasingly popular (see e.g. Charalambous et al. 2020), may be increasingly normalised due to the Covid-19 pandemic, and are expected to gain even more relevance in the near future (Kearns et al. 2010; Millenson et al. 2018). Most importantly, they are often advertised with the term empowerment, e.g.:

- SkinVision,^1^ skin cancer: ‘Empower yourself. Check for skin cancer today’ and ‘Empower and control your health’
- BrainTest,^2^ mild cognitive impairment: ‘a simple, at-home cognitive screening service that can empower and educate’
- Mind Diagnostics,^3^ mental health conditions: e.g. ‘The hope is that this test will help empower you to get the help you need for your addiction’
- Mimi Hearing Test,^4^ hearing impairments: ‘a simple goal: to empower you to hear as well as possible’

Promises of empowerment have received analytic attention in the context of general mHealth, but we assert that self-testing apps take a special place in the mHealth landscape. They deliver information neither irrespective of a diagnosis (like fitness or diet apps) nor to already diagnosed people (like disease management or treatment apps). Rather, they provide an indication (not an official diagnosis^5^) of having a disease, which, according to the advertisements, can allay worry (in case of a negative result) or result in a recommendation to see a doctor (in case of a positive result). If users test positively, this knowledge will, or so is the idea, lead to an earlier diagnosis (Rat et al. 2018), which might enable lifestyle changes, prevent disease development, or make treatment more effective (Kearns et al. 2010). Hence, the empowering potential of self-testing apps might lie in the provision of information previously reserved for a professional healthcare context, which may inform health decisions. It has also been argued that self-testing apps, like mHealth apps, can empower ‘vulnerable and underserved’ communities that do not have access to affordable healthcare (Mechael 2009; Cvrkel 2018). On the other hand, the potential of mHealth apps to empower their users has been questioned with regards to their scientific accuracy (Charalambous et al. 2020; Chan et al. 2021), the shift of responsibilities to individuals (Lupton 2013a; Lupton and Jutel 2015), and the paternalism inherent in health promotion (Morley and Floridi 2020).

Our first aim is to analyse to what extent self-testing apps can empower their users. For this analysis, we first approach the variety of empowerment definitions by categorising literature on empowerment in three levels: (a) general healthcare, (b) mHealth and (c) self-testing (Sect. 2). In doing so, we do not repeat earlier efforts to create clarity within the conceptualization of empowerment (e.g. Starkey 2003; Tengland 2007, 2008; Fumagalli et al. 2015; Agner and Braun 2018) but notice how, despite varying defining attributes (Holmström and Röing 2010), certain elements, namely knowledge and control, are repeated throughout these different levels.

These elements are also prominent in two paradigmatic, general, and often cited definitions by the World Health Organization (WHO), which we use as the basis for our analysis in Sect. 3. Together, these definitions understand empowerment as a process in which patients are given the knowledge and skills to gain greater control over decisions and actions affecting their health in an environment sensitive to community and cultural differences (World Health Organization 2009, 2021). We contrast the control and knowledge described by the WHO definitions with the self-testing apps’ function and demonstrate how self-testing apps may only partly facilitate such control and knowledge.

This analysis enables us to pursue our second aim, which is to problematize a shift in the meaning of empowerment in the context of self-testing and mHealth. This shift, we observe, replaces process-oriented formulations (as found in the WHO definitions) with more goal-oriented formulations which are present in self-testing app literature and advertisements. In Sect. 4, we argue that the goal-oriented formulations reveal a lack of awareness for relational and environmental factors that *are* present in process-oriented formulations. Such factors, which we believe public health and mHealth scholars should be aware of, determine whether self-testing app users can make meaningful, informed health decisions, and, ultimately, the empowerment potential of self-testing apps. We argue that literature and communication around self-testing apps should return to a process-oriented perspective as is prominent in general health empowerment literature. Lastly, we provide several suggestions for how the relational and contextual factors highlighted by a process-oriented version of empowerment could be considered in self-testing apps, and how this could make them more empowering.

## (Mobile) health empowerment

This section demonstrates how the concepts of knowledge and control are interwoven in the debate on empowerment on three levels: health/ patient empowerment in general, digital and mHealth empowerment, and self-testing app empowerment.

### Health/ patient empowerment

The concept of health empowerment has been a topic of discussion since the 1970s (Lupton 2013a) and gained popularity in the 1990s (e.g. Funnell et al. 1991; Gibson 1991; Skelton 1994; Feste and Anderson 1995). Scholarship on health empowerment traditionally focuses on the political dimension of power relations in healthcare, which have been described as disadvantageous particularly for patients (e.g., Roberts 1999). Health empowerment is then predominantly understood as *patient* empowerment, which means that it foregrounds a power shift from healthcare professionals and systems to patients. This process is captured by a widely cited WHO definition (henceforth WHO 1):

WHO 1: Patient empowerment is a process in which patients understand their role, are given the knowledge and skills by their health-care provider to perform a task in an environment that recognizes community and cultural differences and encourages patient participation (World Health Organization 2009).

In line with the notion of a power shift, this definition identifies the healthcare provider as the party in power, who then provides the ‘knowledge and skills’ to the patients. In other words: ‘What needs to happen [for empowerment] is for doctors to come down off their pedestal and for patients to get up off their knees’ (World Health Organization 2012). As the second part of this quote indicates, this process of empowerment being ‘given’ to patients is only part of it; patients also take the role to proactively ‘get up off their knees’.

In the literature, descriptions of this more active patient role often repeat the ‘knowledge and skills’ from WHO 1, e.g.: ‘Patients are empowered when they have knowledge, skills, attitudes, and self-awareness necessary to influence their own behavior and that of others in order to improve the quality of their lives’ (Funnell et al. 1991, p. 38). Knowledge and skills are also strongly connected to the idea of ‘expert patients’, who can work in partnership with healthcare professionals (Shaw and Baker 2004, p. 723): ‘Expert patients […] are those who can manage their own illnesses and conditions by developing knowledge relevant to maintaining health and countering illness’ (Fox et al. 2005, p. 1299).

As the quotes above already suggest, literature on the empowered patient engages many more concepts than knowledge and skills, e.g. responsibility, autonomy, attitudes, and experience. Patients are enabled and expected to ‘define their goals, take responsibility for their medical treatment and increase their autonomy’ (Feste and Anderson 1995; cited in Varekamp et al. 2009, p. 399). It has been argued that such ‘patient participation’ (WHO 1) leads to better health outcomes (Roberts 1999, p. 87; Lu et al. 2018; McCarron et al. 2021; Hickmann et al. 2022). However, increased autonomy and setting goals do not mean that patients do everything by themselves: patient participation means that these shared activities are accompanied by healthcare professionals (Roberts 1999), within a facilitating environment.

During such collaborative decision-making, patients will decide differently due to their personal circumstances. This awareness of situated differences is also reflected in WHO 1, which stresses an ‘environment that recognizes community and cultural differences’. Such differences may play a role in how much empowerment can be achieved, as ‘empowerment efforts must be customised to different patient groups. In some circumstances, empowerment may provide greater benefit to those who are well-educated and better off’ (Angelmar and Berman 2007). This quote emphasises that patients are not equally ‘non-empowered’ to begin with and that care must be taken to not further enhance the power differences between patients who are well-educated and socio-economically privileged, i.e. are more likely to have ‘knowledge and skills’, and those patients who are less so.

### Digital and mHealth empowerment

In mHealth literature, we observe a different focus in the use of the term empowerment. Some literature still focuses on patient empowerment (Lucivero and Jongsma 2018; Faiola et al. 2019), as some mHealth technologies are introduced in a clinical setting. Notably though, mHealth empowerment can be detached from such a care context, as the app *users* are not necessarily *patients* (yet). As mentioned in the introduction, a big part of mHealth apps is directed at people who do not have a diagnosis and consult their apps for fitness programmes, diet advice, or self-tracking. This target group puts many mHealth apps in the realm of health promotion, where empowerment has been defined in the influential WHO health promotion glossary since 1998 (hereafter WHO 2):

WHO 2: In health promotion, empowerment is a process through which people gain greater control over decisions and actions affecting their health (World Health Organization 2021).

Although it shares the characterization of a process, this definition differs from WHO 1 in interesting ways. First, the source of empowerment is not the healthcare provider (as in WHO 1); it remains unclear, and it is not specified how people can ‘gain’ control over decisions and actions. The focus lies less on a power shift from healthcare professionals to users but more on the idea that, in line with a self-testing advertisement mentioned in the introduction (‘Empower yourself!’, ‘Control your health!’), mHealth apps may allow users to empower *themselves* - to ‘get up off their knees’ (World Health Organization 2012). They would do so by providing their users with an unprecedented amount of health information and encouraging them to manage their health proactively (Morley and Floridi 2020). This line of reasoning has also been followed in the context of other mHealth apps directed at diagnosed patients, which help with tracking symptoms or following treatment plans (‘Take control over your disease and health’,^6^ ‘It’s YOUR Health. Take Control’^7^). Through the availability of information, users would be empowered to make rational and reasoned decisions about what to do next (Lupton 2013a, cited in Morley and Floridi 2020, p. 1162). As a result, they should have more control over their health (Lupton 2013b, p. 260).

Control also plays a role in a second difference between WHO 1 and WHO 2. The emphasis no longer lies on knowledge and skills but on control over decisions and actions. An increased control over decisions has also been formulated as an increase in autonomy (Schmietow and Marckmann 2019) or as gaining mastery of one’s own health and life (Tengland 2007, p. 201; Nordgren 2013, p. 259). These concepts may be connected, but it is not clear how the information provided by the apps may lead to knowledge, and how exactly such knowledge leads to control and empowerment (Morley and Floridi 2020).

### Self-testing app empowerment

Although empowerment is a frequently recurring concept in the rhetoric of self-testing apps, scholarship on empowerment in this context is scarce. Since self-testing apps are part of the mHealth realm, they do share potentially empowering features with mHealth apps in general: self-testing apps may empower their users with information and active health management (Morley and Floridi 2020), and benefit ‘vulnerable and underserved populations’ (Cvrkel 2018).

In addition to what other mHealth apps offer, self-testing apps provide a *particular kind of information*, namely information about one’s current health status. Such ‘diagnostic’ information was previously only obtainable through professional healthcare and may carry profound meaning for a user’s self-understanding and future. In this sense, self-testing apps may provide information necessary for the ‘knowledge and skills’ mentioned in WHO 1.

Second, users have control over the way they receive this diagnostic information, i.e. where, when, and in whose presence, which has also been put forward by app providers’ advertisements: ‘Taken from the comfort of your own home. No appointment needed’ (BrainTest). Especially people who are worried about conditions that carry a stigma might appreciate this discretion. This lowers the threshold for testing and may give more control over further health decisions and actions (WHO 2).

A different way to understand self-testing apps as empowering stems from their use in cooperation with the healthcare system. Returning to the idea of a power shift (WHO 1), self-testing apps could provide concerned users with more grounds to consult their doctor. Bringing a positive test result might make them feel more confident about voicing their concerns than showing up ‘empty-handed’. In this case, the test is a way for the patient to ‘get up off their knees’ in a shared decision-making process with their healthcare professional.

## Empowerment through knowledge and control?

In this section we explore to what extent the elements of knowledge and control can be achieved in self-testing apps.

### Empowerment and knowledge

If empowerment involves acquiring ‘knowledge and skills’ that can be utilised to ‘perform a task’ (WHO 1), then, in order to be empowering, self-testing apps need to provide that kind of knowledge to their users. In this section, we show that receiving ‘diagnostic’ information does not necessarily result in such knowledge.

The difference between information and knowledge is well-discussed in information systems literature (e.g. Baskarada and Koronios 2013). Information is described as ‘data that have been shaped into a form that is meaningful and useful to human beings’ (Laudon and Laudon 2006, p. 13; cited in Baskarada and Koronios 2013). Knowledge, on the other hand, is described as ‘the combination of data and information, to which is added expert opinion, skills, and experience, to result in a valuable asset which can be used to aid decision making’ (Chaffey and Wood 2005, p. 223, cited in Baskarada and Koronius 2013). Knowledge is thus a processed and organised ‘next step’ of information. The difference between information and knowledge is functional: while the former is contained in descriptions, the latter is conveyed by instructions and answers to how-to questions (Ackoff 1999). Such instructions, or decision-making aids, are what WHO 1 seems to allude to in its demand for the ‘knowledge and skills […] to perform a task’.

Self-testing apps undoubtedly provide their users with information in the form of a test result. They usually provide a numerical score that is linked to an indication or just a generic recommendation, e.g. to see a doctor or to repeat the test later, but not with other concrete advice. While such descriptive information means something (i.e. is not just data) and may be useful to the app user or others, it does not necessarily provide the actionable knowledge WHO 1 refers to in its definition.

This difference between information and knowledge becomes especially clear in the case of a positive test. A positive result, as information, may lead to an earlier diagnosis, which can leave more room for treatment options, and hence more opportunities for decision-making. Yet in the absence of ‘added expert opinion, skills, and experience’ (Chaffey and Wood 2005, p. 223, cited in Baskarada and Koronius 2013), it does not necessarily provide instructions or contextualization.^8^ On the contrary, a positive result creates many uncertainties about what to do. Could the test result be false positive? What does ‘indication’ mean? Do I really have a disease? Is it serious? What does this mean for my future? Is there anything that can be done about it? Whom should I talk to now? As Swallow summarises:

Paradoxically, the conditions of uncertainty in which early diagnosis is promoted produces, rather than sorts, a number of uncertainties, particularly around patient futures (Swallow 2016).

These uncertainties are especially grave because diagnostic information, albeit only in the form of an indication, is not experienced like any other piece of information; ‘the person is not a neutral spectator when receiving information like a health care diagnosis’ (Kearns et al. 2010, p. 206). For such non-‘neutral spectators’, a possible diagnosis entails a ‘huge psychological dimension’ (Swallow 2016, p. 128) and the need to interpret and articulate the significance of such information (Kearns et al. 2010, p. 206). Even in the case of an indication, i.e. a less ‘final’ result, a new future, in which official diagnoses need to be obtained and decisions about therapies need to be made, could become reality.

With self-testing apps, such uncertainty about the future might be fuelled by conflicting impressions regarding the status of the provided information. While advertisements stress the importance of the test, i.e., assign meaning to the result, their terms and conditions also show disclaimers that clarify that the app is not a medical product and does not provide medical information.^9^ A user’s uncertainty about the result also stems from the fact that many available mHealth apps, including those for self-testing, are of questionable scientific quality (de la Vega and Miró 2014; Charalambous et al. 2020). Even if self-testing apps have been scientifically validated, they cannot avoid false positive and false negative results. Users and patients may not be in a position to appraise the scientific value of test results, which creates uncertainties as well as the need for contextualization.

Uncertainties may be less problematic for the ‘expert patients’ mentioned in Sect. 2, who already have expertise and knowledge at their command, but they are very much present for self-testing app users, who, in the absence of a diagnosis, are less likely to have the same resources. These uncertainties make it difficult to decide what to do next, or in other words, to develop the answers to ‘how-to’ questions that characterise *knowledge* in the sense of WHO 1.

Although self-tests are independently accessible and easy to take, they require contextualization or explanation, often from healthcare professionals or family. It is not a given that patients or users can independently utilise information to generate empowering knowledge, i.e., actionable knowledge that will help to meaningfully engage with a healthcare professional, nor the patient skills required to act on this knowledge and take on an active role.

In sum, receiving a test result can be a first step *towards* the development of this kind of knowledge. However, neither the act of taking a self-test, nor the bringing of a self-test result to a doctor, is necessarily equivalent to taking on the role of the knowledgeable patient who actively cooperates in decision-making.

### Empowerment and control

As explained in the second section, the mHealth discourse often links knowledge to an idea of control (Lupton 2013b). The following section examines to what extent self-testing apps can provide control as described in WHO 2.

As discussed before, self-testing apps provide control over the circumstances in which the test is performed. Deciding and acting upon the questions if, when, and where to take the test could be interpreted as ‘decisions and actions affecting health’ (WHO 2), and having control over said decisions and actions may be, following WHO 2, considered empowering. In this sense, we agree that self-testing apps empower their users to take the test in the circumstances they prefer. The apps might also go beyond the test itself insofar as they, as mentioned in Sect. 2, help users gain the confidence to bring their concerns to a healthcare professional. It also may motivate users to make ‘better’ health decisions. However it is important to note that control over the decision to take the test is *not* control over health or directly linked to a better health outcome.

This missing link may not be problematic were not the control *over test circumstances* being misconstrued as control *over health* when testing apps are advertised (e.g. ‘Control your health’^10^). The advertisements suggest that health is something that *can* and *should* be controlled (see also Sharon 2017, p. 97). In this sense, self-testing apps

represent the vagaries of human embodiment as amenable to control if sufficient vigilance and self-responsibility are exercised on the part of lay people (Lupton and Jutel 2015, p. 132).

It is no coincidence that WHO 2 reads ‘control over health decisions’, not health itself. Although preventive measures are important, full control over health is impossible. This unrealistic idea has consequences for the users. First, it might manifest as an illusory sense of control, which, as has been argued, should not suffice for empowerment (Tengland 2007, p. 201). Second, being in increased control over one’s health also signals increased responsibility over it (Harris, Wathen and Wyatt 2010, p. 223; Kearns et al. 2010). If you are (or feel like you are) in control, you are not only able to act, you (feel that you) are expected to (Kayser et al. 2019). In other words, more control may entail ‘increased obligations and expectations on individuals to take this active role, requiring increasing skills in terms of self-education’ (Nuffield Council on Bioethics 2010).

Self-testing apps often encourage users to self-test regularly, to stay informed, to stay in control. This continuous engagement with the apps is in the commercial interest of app companies and may lead to a dependency on the technology: If the technology is the way to become empowered and stay in control, it becomes indispensable. In this situation of continuous use, in which users are in a perpetual state of concern about their health, monitoring themselves for any changes, and experiencing a pressure to act, users might turn into ‘pre-patients’ (Egher and Wyatt 2016). While acknowledging technology’s potential to help with early diagnosis and treatment, experts worry that the increased availability of vast information could send ‘the “worried well” into hyperdrive’ by causing ‘unnecessary alarm’ (Royal College of Surgeons of England 2018). The combination of overwhelming information and high expectations has been criticised as unhelpful (Angelmar and Berman 2007, p. 155; Lupton 2013a, p. 397), as it sets people up for failure and may ultimately result in blame. This ‘correlative vice’ of empowerment narratives

can feel like an elaborate mechanism for victim-blaming that denies the fact that much of health is controlled by macro forces over which the “user” has only very marginal or no control (Morley and Floridi 2020, p. 1166).

A user’s difficulty in meeting the expectations stemming from empowerment narratives also points to important differences regarding users’ different ‘starting points’. Not all users will be able to manage the responsibility to ‘control their health’ in the same way. Users are, so to speak, not equally ‘non-empowered’ before they start using these apps. As mentioned in Sect. 2, literature on the patient empowerment level has already observed that empowerment does not help patients equally, and that educated and socio-economically privileged users might benefit more from empowerment efforts. In the case of self-testing apps, handling information and responsibility may also require a certain level of ‘pre-existing empowerment’, e.g. a certain level of cognitive capacities (Egher and Wyatt 2016, p. 154), digital literacy, and health literacy (Kreps 2017), as well as material means like access to a smartphone or tablet. Here again, the difference in expertise between expert patients and pre-patient app users comes into play. When considering such individual and contextual conditions, it seems that although nobody can fully control their health, some can more than others. All users are dependent on the means available to them, especially the local healthcare system: Do users have (affordable) access to it? Will the doctor take the user and the test seriously? Does the healthcare system offer post-diagnostic care? In this sense, self-testing apps might promise empowerment to users who do not have the means to empower themselves and who may ultimately be blamed for failing to do so.

## Revisiting empowerment as a process: a strategy

Section 3 demonstrated that the goals of actionable knowledge and control over health decisions can neither be attained by the individual user nor provided by the self-testing app alone. While these shortcomings make self-testing apps less empowering than promised, they also point to an important implication in the meaning of empowerment. Self-testing apps seem to promise (and demand) an end result (‘Control your health!’), a *state* of ‘having knowledge’ and ‘being in control’ through the test result. Such an envisioned *state* of an individual or group aligns with definitions of empowerment as a *goal* in contrast to a *process* (Tengland 2007, 2008; Nordgren 2013). While the WHO definitions formulated patient empowerment as a process, literature on mHealth and self-testing app rhetorics shifted towards presenting empowerment as a goal - some in the form of knowledge and control, some as empowerment as a goal in itself. It is this shift from process to goal that entails the apps’ shortcomings: in the former case, knowledge and control are not fully attainable by the app user and in the latter case, empowerment is mobilised as a positively connotated marketing trope, while its benefits to the user remain unclear (Segers and Mertes 2022, p. 852).

In this section, we argue that a process-understanding of empowerment should guide the conceptualization of empowerment in the context of self-testing apps. Many authors have written about empowerment as a process (Gibson 1991; Rissel 1994; Rodwell 1996; Kabeer 1999; Kayser et al. 2019). Defined as such, empowerment refers to the *means* required to attain the goals sought, which ‘has to do with the means of working toward health, empowerment and quality of life’ (Tengland 2013, p. 143). Two of such means have been formulated within the health empowerment definitions described in Sect. 2, namely (1) cooperation between user/patient and healthcare professionals or other professional expertise, and (2) consideration of individual circumstances of the user/patient when setting goals.

These means are especially valuable for tackling the shortcomings of self-testing apps we identified in Sect. 3. The first one, cooperation with healthcare professionals, points to the expertise and skills necessary to contextualise and operationalise the information provided by the app – or, in other words, to turn information into knowledge. Highlighting a collaborative process relieves users of unrealistic expectations to achieve health goals by themselves, or to be in control of their health altogether. Hence, a process-oriented understanding of empowerment underscores the importance of cooperation and, more widely, the doctor-patient relationship, which allows for patients to receive and discuss the contextualised knowledge and control that can be considered empowering. Understanding empowerment as a process, in this way, also creates room for discussing the effects of self-testing apps on the doctor-patient relationship (for such a discussion, see e.g. Segers and Mertes 2022), which, in an empowerment-as-goal formulation, might be overlooked.

Second, considering a user’s individual circumstances would mean supporting many different end users, with various levels of ‘pre-empowerment’, as well as different intended uses they may have for the apps. Users may not aspire to have ‘control over health’ but may be looking for a way to approach their healthcare professional in a more informed manner. In a similar vein, they may be looking for a way to feel like they are ‘doing something’ to be more involved in their own health, or to open up a dialogue with loved ones about a condition they experience but struggle to describe.

Returning to process considerations of empowerment in the context of self-testing apps can yield several strategies to make these apps more empowering. First, the need for actionable knowledge points to the importance of providing users, as well as others in their social circle, with additional resources to interpret and act upon their test results. In line with what some self-testing apps have begun to offer, these could be video materials with guidance on how to understand what a test outcome means, forums to discuss experiences with others, or materials on ways to support someone who receives a test result. Similarly, users could be supported in their ‘next steps’: Which healthcare professional are located in their region? If avoiding stigma is important to the user, which healthcare professionals are sympathetic to that concern? If none are nearby, what alternatives are available? Which doctors offer video consultations?

A second strategy could be to promote education and support for care providers rather than users. What do healthcare professionals need to know about self-testing technologies, in order to adequately respond to patients who contact them about their results? What do healthcare professionals need to adequately value the outcome of a self-testing app? How do we include healthcare professionals in the development of these technologies?

Finally, we suggest extending the possibility of participation in decision-making from the clinical interaction to the development of health technologies, i.e. users receiving the opportunity to collaborate on the development of self-testing apps. With a process-oriented form of empowerment, it would be considered empowering if users had a seat at the table during the development process and, therefore, were empowered to not just make health decisions, but design the very possibilities for making health-decisions through the app. Such participation would also ensure that self-testing apps best facilitate what later users need to acquire the knowledge and skills central to the empowerment process. In sum, it would give users the chance to be active decision-makers instead of “passive targets” of health technologies (Lupton 2013b).

The strategies presented here are not exhaustive but may provide app developers and public health experts with directions to ensure that self-testing apps are as empowering as possible.

## Conclusion

As self-testing apps offer potentially significant outcomes to their users, it is important to address their empowerment promises. This new and emerging technology is presented with the intuitively attractive goal of facilitating an empowered role for its users. However, what exactly this means, why it is valuable, or how it is to be achieved, remains vague. We observed that this technology is being presented as inherently empowering to the individual user, and have argued that this is a one-sided and incomplete representation. Hence, our position problematises the idea that through the use of technology, empowerment is achievable without relying on healthcare professionals (e.g. Hickmann et al. 2022).

As we have shown, self-testing apps’ functions do not seamlessly fit with the empowerment definitions found in healthcare, nor with those found in health promotion. These apps may, to some extent, empower their users by providing them with control over the test circumstances. However, in this goal-focussed formulation of empowerment, the step from receiving information to taking control, to knowing what to do or decide, or to becoming empowered, is not as immediate or independently achievable as promised.

Attention to the details in process-focussed versions of empowerment can yield strategies for self-testing apps to approximate the knowledge and control that could be considered helpful for making health decisions. With its focus on interaction between app, user and healthcare professional, a process-focused version allows us to consider relational and contextual factors that goal-focussed versions fail to take into account. This focus on interaction also reveals that self-testing apps could be considered empowering beyond the criteria of knowledge and control, as they could increase a user’s self-confidence in approaching a doctor with evidence of their symptoms.

In sum, with a goal-focused sense of empowerment, self-testing apps could be considered to promise much more empowerment than they deliver, and with a process-focused sense of empowerment, the apps could constitute the first step in the empowerment process. With this analysis, we refrain from settling on a particular definition of empowerment for measuring the empowerment facilitated by self-testing apps. It is possible that a new, hitherto undefined sense of empowerment should be used to evaluate self-testing apps, or that empowerment is not the right term for what self-testing apps should aim for.

This article contributes to literature on digital health technologies, which, so has been argued, has been dominated by a ‘largely uncritical preventive medical or health promotion perspective’ (Lupton 2013b). It also adds to the literature on the definition of empowerment (Skelton 1994; Tengland 2007, 2008; Agner and Braun 2018; Morley and Floridi 2020) by showing what is at stake with a goal- or process definition in mHealth.

In discussing the coherence of the term empowerment in the context of self-testing, we did not engage with user experiences of empowerment. It is unclear to what extent such experiences should be accounted for in definitions of empowerment (Tengland 2007, p. 201) but such qualitative future research could benefit the debate by potentially describing new, unexpected ways and criteria to be empowered. Hence, additional research is needed to increase our understanding of empowerment in the context of self-testing apps. What do users experience as empowering features of self-testing apps, for example? What do they need? And why do they need an app for that? Such research is beyond the scope of this article. We hope, however, that the considerations presented here and the demonstrated importance of procedural elements can provide a first direction in pursuing such efforts.

## Footnotes

1 https://www.skinvision.com.

2 https://braintest.com.

3 https://www.mind-diagnostics.org/get-the-app.

4 https://mimi.health/hearing-test-apps.

5 See for example the disclaimers by SkinVision https://www.skinvision.com/terms/ and BrainTest https://braintest.com/terms/.

6 Happi app, https://happiapp.nl.

7 Vilua app, https://vilua.com.

8 Some self-testing apps additionally explain the result in the form of a pre-recorded video. Although expert opinion has been part of making these videos, they are still generic and recorded without any knowledge about the patient themselves. Hence, they may help with contextualisation of what the score means, but not necessarily with what the score means *for the user* and for what to do next.

9 See for example the websites of SkinVision, BrainTest.

10 Skinvision app, https://www.skinvision.com.
